# Aspartimide Formation and Its Prevention in Fmoc Chemistry Solid Phase Peptide Synthesis

**DOI:** 10.1002/cbic.202500490

**Published:** 2025-08-26

**Authors:** Marco J. W. Kong, Tim J. H. P. van den Braak, Kevin Neumann

**Affiliations:** ^1^ Institute for Molecules and Materials Radboud University Heyendaalseweg 135 Nijmegen 6525 AJ Netherlands

**Keywords:** aspartic acid, aspartimide, fmoc chemistry, peptide synthesis, solid phase peptide synthesis

## Abstract

Aspartimide formation remains a persistent challenge during Fmoc chemistry solid phase peptide synthesis. This review outlines various strategies to suppress base‐mediated aspartimide formation, including the use of ester β‐carboxyl protecting groups, non‐ester‐based β‐carboxyl masking groups, and backbone‐protecting groups. In addition, alternatives to the Fmoc group are explored that are cleavable under nonbasic conditions. The work discussed in this review highlights that the continued development of compatible and scalable tactics toward aspartimide prevention is essential for advancing peptide synthesis in both research and industry.

## Graphical abstract

Aspartimide formation remains a persistent challenge in Fmoc solid phase peptide synthesis. This review surveys strategies to prevent aspartimide formation, covering protecting group design, backbone protection, and alternative deprotection approaches. Continued development of scalable, compatible tactics is key to advancing peptide synthesis.

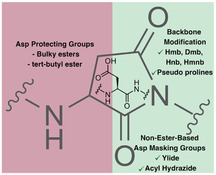

## 1 Introduction

Ever since the Nobel prize‐winning breakthrough of synthesizing peptide segments on solid support by Robert Bruce Merrifield,^[^ ^1^ ^]^ peptide chemistry has seen many advances. Peptides are unique building blocks for the fields of pharmaceuticals,^[^ ^2^, ^3^ ^–^ ^4^ ^]^ cosmetics,^[^ ^5^ ^,^ ^6^ ^]^ supplements,^[^ ^7^ ^]^ and biomaterials.^[^ ^8^ ^,^ ^9^ ^]^ Especially in the pharmaceutical industry, peptide chemistry has seen a rise during the past decades, with more than 50 peptide‐based drugs approved by the FDA since 2010.^[^ ^10^ ^]^ Hence, affordable, efficient, and gram‐scale routes for peptide synthesis are crucial to sustaining such demand. Automated solid‐phase peptide synthesis (SPPS) with the now standardized fluorenyl methoxycarbonyl (Fmoc) strategy has become widely used compared to classical *tert*‐butyloxycarbonyl (Boc) SPPS because of its convenience and the non‐use of volatile and corrosive TFA in N^α^ PG removal.^[^ ^11^ ^]^ The versatility of amino acid building blocks provides many opportunities for new peptides, and the use of peptidomimetics or peptoid strategies further broadens the accessible chemical space.^[^ ^12^ ^,^ ^13^ ^]^ Furthermore, the addition of nucleotides in the form of peptide nucleic acids (PNAs) for antisense therapies^[^ ^14^ ^,^ ^15^ ^]^ and carbohydrates for glycopeptides as a tool for studying post‐translational modifications^[^ ^16^ ^]^ has seen a rise. Although peptide chemistry has advanced considerably,^[^ ^17^ ^]^ the formation of aspartimide (Asi) via side‐chain cyclization of aspartic acid continues to hinder the synthesis of extended peptide sequences by the almost universally used stepwise Fmoc chemistry SPPS technique.

**Figure 1:**
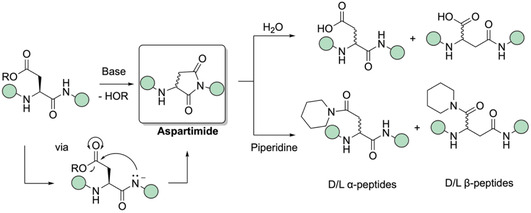
Base‐catalyzed aspartimide formation, followed by nucleophilic attack on Asi, results in racemization and α‐ and β‐peptides.

The earliest report on Asi formation dates back to 1888, when Piutti investigated α‐ and β‐asparagines. However, during the reaction of aspartic acid in hot alcoholic ammonia, Piutti was unable to isolate the expected diamide.^[^ ^18^ ^]^ Instead, the reaction yielded a cyclized side product, nowadays widely recognized in peptide chemistry as aspartimide. These days, aspartimide is typically encountered during standard Fmoc‐deprotection by treatment with 20% piperidine (**1**) in DMF. Deprotonation of the C‐terminal backbone amide of the Asp residue results in nucleophilic attack at the carbonyl of the β‐carboxyl. Importantly, reversible rehydration of the formed imide results in racemization and formation of a mixture of α‐ and β‐peptides.^[^ ^19^ ^]^ Additionally, piperidine (or any other nucleophilic base) reacts with aspartimide to form α‐ and β‐base adducts (**Figure** **1**). These side products not only lower the yield, but are notoriously difficult to separate, or could even entirely prevent the formation of the desired peptide product. A major challenge is to seperate D/L‐β‐aspartyl peptides which are typically formed in a 3:1 ratio compared to the α‐aspartyl peptide.^[^ ^20^ ^]^

The propensity of Asi formation depends on the nature of the C‐terminal amino acid residue linked to Asp, whose backbone amide nitrogen acts as the nucleophile attacking the Asp side chain to form the five‐membered ring. The formation is especially prevalent in Asp‐Gly motifs, likely because Gly is the least sterically hindered residue.^[^ ^21^ ^]^ For this reason, research on the prevention of Asi often uses model peptides displaying the Asp‐Gly motif.

In 1987, residues 22–27 of the adrenocorticotropic hormone (Val‐Tyr‐Pro‐Asn‐Gly‐Ala) were used to monitor succinimide‐related reactions.^[^ ^22^ ^]^ In this case, deamination of Asn made the sequence prone to Asi formation.^[^ ^22^ ^]^ Karlström et al. later used the sequence, with Asn replaced by Asp, to evaluate Asp protecting groups aimed at preventing Asi formation.^[^ ^23^ ^]^ Another widely used Asi‐prone sequence is the small fragment of the scorpion toxin II species *Androctonus australis Hector* (Val‐Lys‐Asp‐Gly‐Tyr‐Ile).^[^ ^21^ ^,^ ^23^, ^24^ ^–^ ^29^ ^]^ This sequence was, for example, used by Lauer et al. to study the effect of the C‐terminal residue linked to Asp on Asi formation during Fmoc‐SPPS. Together with others, the authors determined that besides Gly, in particular Ala, Ser, Thr, Cys, Arg, Asp, and Asn, the C‐terminal residues of Asp display especially Asi‐prone motifs.^[^ ^30^ ^,^ ^31^ ^]^ Lauer et al. also found that even in the presence of bulky protecting groups, such as Arg(Pmc), Cys(Amc), and Asn(Trt), Asi formation occurred.^[^ ^30^ ^]^ The role of the side‐chain protecting group of the residue following Asp was further highlighted by M. Mergler et al., who observed that the motif Asp(OtBu)‐Cys(Acm), as part of scorpion toxin II, resulted in 27% Asi formation upon prolonged basic treatment, whereas Asp(OtBu)‐Cys(Trt) yielded only 5.5% Asi under otherwise comparable conditions.^[^ ^26^ ^]^

While sequence context, particularly the identity of Xx in Asp‐Xx motifs, plays a major role in Asi formation, factors such as peptide conformation, solvent, temperature, and the base used for Fmoc‐deprotection also significantly influence the extent of this side reaction.^[^ ^32^ ^]^ In this context, small amounts of water are reported to cause additional instability of the corresponding Asp(OtBu) residue.^[^ ^33^ ^]^ The polarity of the solvent has a strong influence on Asi formation, with higher polarity leading to more Asi.^[^ ^32^ ^]^

Although most commonly observed during Fmoc‐deprotection, Asi formation can also occur in various late‐stage peptide modifications. For example, Lansbury and coworkers were investigating synthetic *N*‐glycopeptides in which, upon activation of the aspartic acid residue, aspartimide was formed rather than the desired *N*‐linked glycopeptide.^[^ ^34^ ^]^ Furthermore, when coupling peptide segments utilizing chemoselective peptide bond‐forming reactions such as the native chemical ligation and bis(2‐sulfanylethyl)amido ligation, the presence of Asi was also observed under forcing conditions of elevated pH and temperature.^[^ ^35^, ^36^, ^37^ ^–^ ^38^ ^]^

Asi formation remains a formidable challenge in Fmoc chemistry SPPS, resulting in a side reaction which is often unpredictable and causes time‐consuming and costly purification steps in both industry and academic research.^[^ ^39^ ^]^ This review surveys recent advances in understanding and preventing Asi formation in Fmoc peptide synthesis, with the aim of guiding future synthetic strategies and research directions.

As a result of the persistent challenge of Asi formation during Fmoc chemistry SPPS, multiple strategies were reported to minimize Asi formation, including the use of ester‐protecting groups on the β‐carboxyl of Asp (Chapter 2). However, only the use of non‐ester‐based aspartic acid side chain masking groups and backbone protection has been shown to fully eliminate the formation of Asi (Chapter 3). In addition, new approaches for α‐amine protecting groups and modulation of the basicity of the Fmoc‐deprotection condition are a viable strategy (Chapter 4).

## 2 Minimizing Aspartimide Formation with Ester‐Based β‐Carboxyl Protecting Groups

Currently, the protecting group of choice for the Asp side chain is the *tert*‐butyl ester (OtBu).^[^ ^40^ ^,^ ^41^ ^]^ However, its relatively low steric hindrance can cause complications in Asi‐prone sequences. Bulkier protecting groups have been evaluated as a strategy to mitigate Asi formation, and their performance will be discussed in detail in this article; however, increased steric demand does not necessarily ensure effective Asi suppression. In fact, trityl and ortho‐esters unexpectedly promote Asi formation^[^ ^25^ ^]^ despite their prevention on other intramolecular cyclization reactions such as diketopiperazine (DKP) formation.^[^ ^42^ ^]^

Historically, Boc‐based SPPS resulted in only minimal Asi formation,^[^ ^43^ ^]^ largely due to the use of Asp(OcHex) side‐chain protection, particularly when combined with in situ neutralization coupling protocols employing DIEA as a sterically hindered base. However, the use of HF during the final cleavage/deprotection step promotes acid‐catalyzed Asi formation.^[^ ^44^ ^]^

The first Fmoc‐compatible Asp side chain protecting group designed to prevent Asi formation was reported in 1987. Okada et al. reported the synthesis of β−1 and β‐2‐adamantylaspartates (**2, 3**, O‐1‐Ada and O‐2‐Ada, **Table** **1**) which were readily accessible from the corresponding side‐chain unprotected Asp using an esterification protocol with DCC and DMAP. These protecting groups can be cleaved with TFA, making them suitable for Fmoc‐SPPS.^[^ ^45^ ^,^ ^46^ ^]^ The exponential use of Fmoc‐SPPS during the 1990s resulted in additional new protecting groups for the β‐carboxyl of Asp, namely 2,4‐dimethyl‐3‐pentyl (**4**, ODmp)^[^ ^47^ ^]^ and 3‐methyl‐3‐pentyl (**5**, OMpe) esters (Table 1).^[^ ^23^ ^,^ ^26^ ^]^ The latter was installed using oxalyl chloride to form a reactive acyl chloride, which was more efficient than standard DCC and DMAP esterification protocols.^[^ ^23^ ^]^ These protecting groups are sterically demanding, similar to 1‐ and 2‐adamantyl esters. Notably, Karlström proposed that the flexibility of OMpe contributes to a further reduction in Asi formation, as it interferes with the ability of the deprotonated backbone amide to attack the β‐carboxyl group.^[^ ^23^ ^,^ ^26^ ^]^ Indeed, the OMpe protecting group remains one of the most widely used options for suppressing Asi formation and has been employed in numerous successful peptide syntheses, including the ATPase family AAA domain‐containing protein 2 (ATAD2 bromodomain), DARPin pE59, Equinatoxin II 1–85 analog (EqtII 1–85), and microtubule‐associated protein Tau (MAPT).^[^ ^48^, ^49^, ^50^ ^–^ ^51^ ^]^

**Table 1:** Overview of ester‐based protecting groups (2‐10) and non‐ester‐based aspartic acid side chain masking groups (11‐14).

Protecting group	Molecular structure 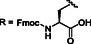	Abbreviation	Formation	Deprotection
O‐1‐adamantyl^[^ ^45^ ^,^ ^46^ ^]^ (**2**)	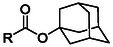	1‐Ada	DCC, DMAP	>90% TFA
O‐2‐adamantyl^[^ ^45^ ^,^ ^46^ ^]^ (**3**)	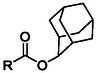	2‐Ada	DCC, DMAP	>90% TFA
O‐2,4‐dimethyl‐3‐pentyl^[^ ^47^ ^]^ (**4**)	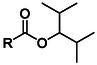	Dmp	DCC, DMAP	>90% TFA
O‐3‐methyl‐3‐pentyl^[^ ^23^ ^,^ ^26^ ^]^ (**5**)	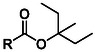	Mpe	Oxalyl chloride	>90% TFA
O‐2,3,4‐trimethyl‐3‐pentyl^[^ ^27^ ^]^ (**6**)	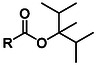	Die	DCC, DMAP	>90% TFA
O‐3‐ethyl‐3‐pentyl^[^ ^29^ ^,^ ^53^ ^]^ (**7**)	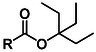	Epe	Cl3CN, NaH	1% TFA
O‐4‐n‐propyl‐4‐heptyl^[^ ^29^ ^,^ ^53^ ^]^ (**8**)	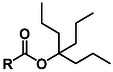	Php	DCC, DMAP	>90% TFA
O‐5‐n‐butyl‐5‐nonyl^[^ ^29^ ^,^ ^53^ ^]^ (**9**)	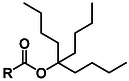	Bno	DCC, DMAP	>90% TFA
O‐2‐phenylisopropyl^[^ ^33^ ^]^ (**10**)	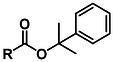	PhiPr	DCC, DMAP	>90% TFA
N‐4‐methoxy‐7‐nitroindoline^[^ ^57^ ^]^ (**11**)	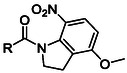	MNI	1) CH_3_I, K_2_CO_3_2) NaBH_3_CN3) DIC4) AgNO_3_	h*ν* (365 nm)
Cyanosulfurylides^[^ ^59^ ^]^ (**12**)	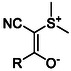	CSY	T3P, DIPEA	NCS
Cyanopyridiniumylides^[^ ^61^ ^]^ (**13**)	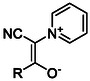	CyPY	T3P, DIPEA	HCl (aq.)
N‐Boc‐Hydrazide^[^ ^62^ ^]^ (**14**)	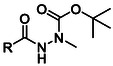	NHNMeBoc	1) Ac_2_O2) Boc‐N_2_H_2_	CuSO_4_ (aq.)

Limitations are reached when attempting to use even bulkier esters, as demonstrated by Mergler and Dick in 2005.^[^ ^27^ ^]^ Their investigation of a protecting group with increased steric demand compared to OMpe resulted in low yields and lengthy synthetic procedures. However, 2,3,4‐trimethyl‐3‐pentyl ester (**6**, ODie) was successfully synthesized after repeated coupling cycles with DCC and demonstrated comparable efficacy to OMpe in preventing Asi formation in the scorpion II model peptide. But its stability in prolonged piperidine and DBU exposure could be beneficial for longer peptides compared to OtBu and OMpe. Successful synthesis of one Asi‐prone segment as part of the human parathyroid hormone (PTH) [hPTH(1–84)] was achieved when replacing OtBu with ODie.^[^ ^52^ ^]^ In contrast to ODie, the significantly bulkier tricyclohexyl methyl (Tcm) version was not accessible even with many esterification conditions.^[^ ^27^ ^]^

Research on extending the alkyl chains even further was conducted by Behrendt et al. The authors accessed aspartic esters with 3‐ethyl‐3‐pentyl (**7**, OEpe), 4‐n‐propyl‐4‐heptyl (**8**, OPhp), and 5‐n‐butyl‐5‐nonyl (**9**, OBno), which showed significantly lower Asi formation with increasing steric demand.^[^ ^53^ ^]^ This confirms the observation that not only does bulkiness affect the Asi formation, but also the flexibility of the alkyl chains. Teduglutide (Gly^2^)‐GLP‐2, which has the problematic motifs Asp^16^‐Asn and Asp^3^‐Gly, was synthesized with Asp(OBno), which resulted in a 25% reduction of Asi formation compared to Asp(OtBu).^[^ ^29^ ^]^

The incorporation of *N*‐ or *O*‐linked oligosaccharides in Fmoc‐SPPS has significantly advanced the field of functional peptide synthesis.^[^ ^54^ ^]^ Since direct incorporation of Asn(Sug) monomers into peptide sequences via Fmoc‐based SPPS is challenging due to the significantly reduced efficiency of peptide elongation, orthogonal Asp protecting groups were investigated to enable late‐stage on‐resin modifications.^[^ ^34^ ^,^ ^55^ ^]^ For example, 2‐phenylisopropyl (**10**, PhiPr) displays more lability toward acids compared to OtBu, which makes cleavage viable with less than 5% TFA.^[^ ^56^ ^]^ The obtained selectivity was employed by Chen and Tolbert, who employed PhiPr as an alternative to O‐allyl protection.^[^ ^33^ ^]^ On‐resin deprotection was carried out with 1% TFA and subsequent glycosylation of aspartic acid with glycosylamines, GlcNAc, or Man_8_GlcNAc_2_. This strategy proved more effective in reducing Asi formation than the orthogonal O‐allyl group and enabled the successful synthesis of the biologically active N‐linked glycopeptide C34‐human immunodeficiency virus entry inhibitor.^[^ ^33^ ^]^

## 3 Fully Eliminating Aspartimide Formation with Non‐Ester‐Based Asp Side Chain Masking Groups and Backbone Protection Group Schemes

### 3.1 The Use of Non‐Ester‐Based Aspartic Acid Side Chain Masking Group

Ester‐based β‐carboxylic acid protecting groups have been extensively studied, but no derivative fully eliminates Asi formation due to the electrophilic nature of esters. Already in 2015, Zheng and coworkers developed an Fmoc‐compatible, photocleavable protecting group displaying an amide bond for Asp and Glu.^[^ ^57^ ^]^ 4‐methoxy‐7‐nitroindoline (**11**, MNI, Table 1) was obtained from a multistep synthesis including methylation, reduction, and nitration. No Asi formation was observed when using this protecting group with model peptide VSDGNG, and photolysis was successfully demonstrated at 365 nm to obtain the native peptide.^[^ ^57^ ^]^ Due to its rapid photolysis kinetics, the MNI group was fully cleaved within one minute. Endothelin‐1 (ET‐1) has been successfully synthesized using MNI, demonstrating its advantage over previously reported photocleavable protecting groups, including 4,5‐dimethoxynitrobenzyl.^[^ ^58^ ^]^

**Figure 2:**
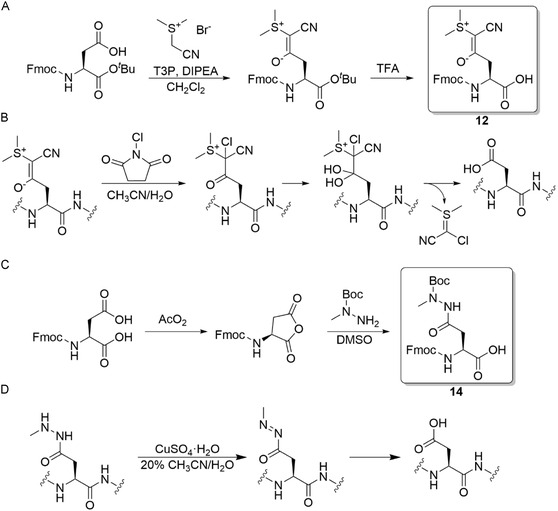
A) synthesis of CSY protecting group B) postulated mechanism of CSY removal C) formation of Boc‐hydrazide protecting group **D)** Postulated mechanism of Asp(NHNMe) deprotection.

An alternative approach has recently been featured by Neumann et al., where a stable C—C bond is employed to mask the inherently more labile C—O bond of the typically ester‐based protecting groups. For this purpose, the authors reported the use of cyanosulfurylides (**12**, CSY, Table 1). These formally zwitterionic species are stable to common SPPS manipulations and, in addition, improve on‐resin solubility. Sulfur ylides can be made from commercially available Fmoc‐Asp(OH)‐OtBu. Bromoacetonitrile and dimethyl sulfide were combined to obtain the corresponding sulfonium salt. Subsequent addition of propanephosphonic acid anhydride (T3P) and N, N‐Diisopropylethylamine (DIPEA) led to obtaining Fmoc‐Asp(CSY)‐OtBu (**Figure** **2A**). While CSY is not cleaved with TFA, the ylide can be chemoselectively removed with an electrophilic halogen source such as NCS within an aqueous environment (Figure 2B). This oxidative deprotection step is compatible with all amino acids except methionine and free cysteine.^[^ ^59^ ^]^

Additionally, the polarity of the CSY group has been shown to be beneficial for preventing on‐resin aggregation, as demonstrated by the group of Thomas by synthesizing difficult β‐sheet peptides which were prone to Asi formation and aggregation.^[^ ^60^ ^]^ Importantly, the Thomas group also reported that, although CSY is stable at room temperature, side reactions may occur at elevated temperatures, particularly during Fmoc‐removal cycles.

Adding onto this research, Bode's group has developed cyanopyridiniumylides (**13**, CyPY, Table 1) which extend the ylide‐based carboxylic acid masking groups from sulfur ylides to pyridinium ylides. In contrast to its sulfur analog, deprotection is carried out in aqueous acidic solution (HCl in H_2_O/DMSO) rather than with an oxidative halogen source, making it compatible with Met and Cys(Acm) residues in SPPS. The authors employed this building block for the synthesis of low‐density lipoprotein receptor class A domains, which display multiple Asp residues. For this purpose, the authors employed KAHA ligation, demonstrating the compatibility of Asp(CyPY) with complex synthetic manipulations and further expanding the toolbox of chemical building blocks for accessing synthetic peptides of high therapeutic interest.^[^ ^61^ ^]^

The CSY protecting group has given rise to new non‐ester‐based Asp side chain protecting groups. In the following years, Mase et al. investigated the use of acyl hydrazides as an alternative protecting group scaffold. Starting from N‐protected aspartic acid, intramolecular anhydride formation was achieved with acetic anhydride, and subsequent ring opening with Boc hydrazine resulted in the Boc‐hydrazide (**14**) (Figure 2C, Table 1).^[^ ^62^ ^]^ The acyl hydrazide can be converted to the free carboxylic acid using copper‐mediated hydrolysis (Figure 2D). For this purpose, an aqueous solution of copper sulfate (CuSO_4_) was employed at 37 °C for 3hr. Asi formation was decreased to <1% in comparison to 7% when utilizing Asp(OtBu) for the synthesis of fluoroacetate dehalogenase with an Asi‐prone Asp‐Arg motif.^[^ ^62^ ^]^ However, the use of copper in peptide chemistry poses challenges due to Good Manufacturing Practice‐related concerns and the tendency of copper ions to coordinate with peptides, potentially leading to purification difficulties and residual metal contamination.

### 3.2 Backbone Protecting Groups

While extensive efforts have focused on optimizing β‐carboxyl protecting groups for Asp, complementary strategies have been developed to mitigate aspartimide formation. As illustrated by Sheppard and coworkers, a nucleophilic nitrogen of the amide is essential for Asi formation. Hence, masking the amide linkage between an Asp residue and its C‐terminally linked residue completely eliminates Asi formation.^[^ ^63^ ^]^ Johnson and coworkers first employed the 2‐hydroxy‐4‐methylbenzyl (**15**) (Hmb, **Table** **2**) group to minimize Asi formation.^[^ ^64^ ^]^ The Hmb protection scheme was used in combination with Asp(OtBu) to synthesize scorpion toxin II model peptide. When the synthesis of the peptide fragment was attempted without Hmb as a backbone‐protecting group, it could only be isolated in low yields, with only 30% being the unmodified peptide; the remainder consisted of aspartimide as well as α‐ and β‐piperidides. In contrast, with the use of Hmb, the toxin II fragment was obtained with 90% yield.^[^ ^64^ ^]^

**Table 2:** Overview of backbone‐protecting groups. Aa = any amino acid.

Protecting group	Molecular structure 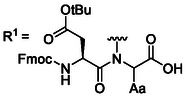	Abbreviation	Coupling	Deprotection
2‐hydroxy‐4‐methoxybenzyl^[^ ^64^ ^]^ (**15**)2‐acetoxy‐4methoxybenzyl^[^ ^68^ ^]^ (**16**)	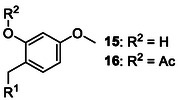	Hmb, AcHmb	Gly and Ala, but as dipeptide for the rest	>90% TFA, AcHmb is TFA resistant
2,4‐dimethoxybenzyl^[^ ^70^ ^]^ (**17**)	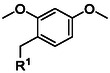	Dmb	As dipeptide	>90% TFA
1‐methyl‐3‐indolylmethyl^[^ ^28^ ^]^ (**18**)	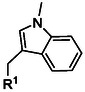	MIM	Tested for Gly, but as dipeptide for the rest	>90% TFA
3,4‐ethylenedioxy‐2‐thenyl^[^ ^28^ ^]^ (**19**)	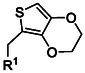	EDOTn	Tested for Gly, but as dipeptide for the rest	>90% TFA
2‐hydroxy‐6‐nitrobenzyl^[^ ^67^ ^]^ (**20**)	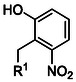	Hnb	Tested for β‐branched amino acids	h*ν* (366 nm)
2‐hydroxy‐4‐methoxy‐5‐methylsulfinyl benzyl^[^ ^78^ ^]^ (**21**)	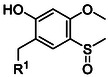	Hmsb	Tested for Ala and Val	TMSBr/EDT/TFA/thioanisole
2‐hydroxy‐4‐methoxy‐2‐nitrobenzyl^[^ ^79^ ^,^ ^80^ ^]^ (**22**)	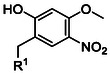	Hnmb	All amino acids	SnCl_2_/CrCl_2_,TFA
Pseudo prolines^[^ ^82^ ^,^ ^83^ ^]^ (**23–25**)	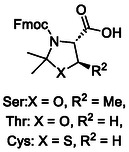	ψMe,Mepro	Dipeptide, but also stand‐alone	>90% TFA

**Figure 3:**
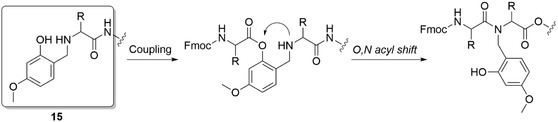
Mechanism of intramolecular O,N acyl transfer, which assists the coupling of amino acids on a secondary amine.

The coupling efficiency of Hmb monomers is significantly enhanced by the presence of the free hydroxyl in Hmb through an internal base‐catalyzed mechanism.^[^ ^65^ ^,^ ^66^ ^]^ First, the 2‐hydroxyl is acylated with a subsequent intramolecular *O*,*N* acyl transfer (**Figure** **3**). For Hmb, such intramolecular acyl transfer was observed when coupled to small residues like Gly and Ala, but with sterically more demanding residues like Phe, Leu, and Val, *N*‐acylation became impossible.^[^ ^66^ ^]^ Likely poor *O*,*N*‐acylation kinetics were the cause for inefficient coupling of larger residues.^[^ ^67^ ^]^

While Hmb alone is readily cleaved with TFA, acetylation of its phenolic group yields 2‐acetoxy‐4‐methylbenzyl (**16**, AcHmb), which is highly resistant to TFA and can only be cleaved after hydrolysis regenerates Hmb.^[^ ^68^ ^]^ The main advantage of late‐stage removal of AcHmb is its ability to increase the solubility of ‘difficult’ peptides, thereby facilitating purification, particularly in cases such as β‐amyloid peptides.^[^ ^69^ ^]^

Derivatives of Hmb include 2,4‐dimethoxybenzyl (**17**) (Dmb) and 2,4,6‐trimethoxybenzyl (Tmb) (Table 2). Dmb was found to have higher solubility, higher coupling efficiency and increased acid lability compared to Hmb.^[^ ^70^ ^]^ However, since Dmb lacks a free hydroxyl group, *O, N*‐acyl migration cannot occur, which renders coupling to Dmb‐protected amino acids more challenging. Consequently, the use of dipeptide building blocks, such as Fmoc‐Asp(OtBu)‐(Dmb)Gly‐OH, is beneficial and enables feasible incorporation of Dmb into peptide synthesis protocols.^[^ ^71^ ^]^ Using dipeptides has proven successful and was employed for the total synthesis of Small Ubiquitin‐like Modifier proteins SUMO2/3.^[^ ^36^, ^72^, ^73^ ^]^

In 2008, Isidro‐Llobet et al. introduced non‐benzyl based backbone‐protecting groups.^[^ ^28^ ^]^ The use of 1‐methyl‐3‐indolylmethyl (**18**) (MIM) and 3,4‐ethylenedioxy‐2‐thenyl (**19**) (EDOTn) as protecting groups was inspired by resin linkers employed in solid‐phase‐synthesis.^[^ ^74^ ^,^ ^75^ ^]^ EDOTn, in particular, showed favorable properties due to its reduced steric bulk compared to Dmb, resulting in improved coupling yields. While MIM was more sterically hindered, MIM was found to be more acid‐labile than Dmb, with no protected peptide being observed in the crude.^[^ ^28^ ^]^ Despite these advantages, once again, efficient coupling was largely limited to glycine, with typically a larger excess (10 eq.) being required. Nevertheless, these backbone‐protecting groups still suggest potential as a dipeptide building block compared to Dmb due to their smaller steric hindrance.

To overcome poor *O*,*N*‐acylation kinetics of Hmb, Miranda et al. sought to improve these kinetics with more electron‐withdrawing groups on the auxiliary. The authors identified 2‐hydroxy‐5‐nitrobenzyl (2,5‐Hnb) and 2‐hydroxy‐6‐nitrobenzyl (**20**) (Hnb) as suitable backbone‐protecting groups that facilitate *O*,*N*‐acylation further and thus, are capable of coupling large and β‐branched residues.^[^ ^67^ ^]^ Incorporation of Hnb was achieved through a reductive amination strategy, wherein the substituted salicylaldehyde forms an imine with the amine, followed by reduction with sodium borohydride. Mild photolysis (366 nm) can be conducted to remove these groups effectively.^[^ ^67^ ^]^ However, photolysis makes the protecting group less suitable for incorporation into standard Fmoc‐SPPS workflows.

With these limitations in mind, Abdel‐Aal et al. developed an improved backbone‐protecting group for SPPS, namely 2‐hydroxy‐4‐methoxy‐5‐methylsulfinyl benzyl (**21**) (Hmsb). Although sulfur‐containing moieties had previously been explored for their electron‐withdrawing properties, earlier candidates such as 6‐hydroxy‐5‐methyl‐1,3‐benzoxathiolyl and 3‐methylsulfinyl‐4‐methoxy‐6‐hydroxybenzyl (SiMB) either proved too stable under TFA treatment or exhibited inefficient coupling during SPPS.^[^ ^76^ ^,^ ^77^ ^]^ Instead, Hmsb was introduced as novel protecting group.^[^ ^78^ ^]^ Notabley, Hmsb was quantitatively coupled to amino acids on‐resin via reductive amination, and subsequent cleavage was achieved using a mild reducing cocktail containing trimethylsilyl bromide (TMSBr) and thioanisole. Coupling efficiency was demonstrated on Hmsb‐protected Ala and even beta‐branched Val residues.^[^ ^78^ ^]^

Interestingly, subsequent work by the same research group discloses 2‐hydroxy‐4‐methoxy‐5‐nitrobenzyl (**22**) (Hmnb), which features a nitro group instead of a sulfoxide. The more electron‐withdrawing nitro residue was expected to enhance *O*,*N*‐acylation kinetics.^[^ ^79^ ^]^ Introduction of Hmnb was automated and carried out on‐resin by the addition of 4‐methoxy‐5‐nitrosalicylaldehyde through reductive amination. On‐resin reduction of the nitro group to an aniline derivative using CrCl_2_ unveils acid‐lability in the presence of TMSBr and thioanisole. The aspartimide‐prone sequence VKDGYL was successfully synthesized using Hmnb.^[^ ^79^ ^]^ Around the same time, Liu and coworkers had independently developed a removable backbone tag that was structurally identical to Hmnb.^[^ ^80^ ^]^ Reductive amination of the removable backbone tag was shown to be suitable for on‐resin chemistry. Upon reduction of the nitro group using SnCl_2_, further modification of the peptide could be carried out, for example, installing a solubility tag. Ultimately, the backbone tag could be removed by hydrolysis of the phenolic acetyl residue with subsequent treatment with TFA. Notably, Hmnb was used for the synthesis of the Asp‐based lactam cyclic peptide A‐183, which was inaccessible without the use of Hmnb as an auxiliary.^[^ ^81^ ^]^

Notably, when the amino acid at the C‐terminus of an aspartic acid residue is serine, threonine, or even cysteine, an alternative strategy for suppressing aspartimide formation becomes viable using pseudo prolines (ψ‐Pro). These temporary oxazolidines and thiazolidines are formed from the side chain hydroxyl or thiol residues of Ser (**23**), Thr (**24**), and Cys (**25**), respectively, and the backbone amide nitrogen.^[^ ^82^ ^]^ Introduction is typically done as a dipeptide building block such as Fmoc‐Aa‐Ser(ψ‐Me, Mepro)‐OH or Fmoc‐Aa‐Thr(ψ‐Me, Mepro)‐OH.^[^ ^83^ ^]^ The primary interest for the use of pseudo prolines is to disrupt local secondary structure and enhance solvation of the growing peptide, thereby reducing on‐resin aggregation and improving overall coupling efficiency. Upon global deprotection and cleavage with TFA, pseudo prolines are quantitatively converted back to native Ser or Thr.^[^ ^82^ ^,^ ^83^ ^]^ In addition, pseudo prolines have been shown to effectively eliminate the formation of Asi when displaying an aspartic residue. Successful syntheses are often performed in combination with backbone‐protected amino acids (Hmb or Dmb) and were employed to access, among others, the FASdeath domain,^[^ ^84^ ^]^ D2 domain of human vascular endothelial growth factor receptor 1 (VEGFR1),^[^ ^85^ ^]^ and ubiquitin (Ub).^[^ ^86^ ^]^ This combination was preliminary used to prevent the aggregation of these difficult sequences. Furthermore, the R2 subunit of ribonucleotide reductase (RNR) was successfully obtained using (Asp(OtBu)‐Ser(ψMe, Mepro)‐OH), in which no Asi was observed.^[^ ^87^ ^]^

The group of Unverzagt employed pseudo prolines for the synthesis of different glycopeptides. The group observed that the formation of Asi during synthesis of *N*‐glycopeptides was significantly reduced when installed in proximity to the Asp residue during both peptide elongation as well as during the subsequent aspartylation.^[^ ^88^ ^]^ Similarly, Wang et al. have demonstrated that pseudo prolines mitigate Asi formation when installed at (*n* + 2) position from Asp, as shown in the successful synthesis of erythropoietin glycopeptide fragments.^[^ ^89^ ^]^ In this case, the formation of Asi has occurred as the Lansbury product during the installation of the glyco‐residue, but was suppressed with the installation of the according pseudo proline.

## 4 Miscellaneous

### 4.1 Fmoc‐Deprotection: Bases and Additives

A major challenge in Fmoc‐based SPPS is the formation of Asi by the base‐catalyzed mechanism. This side reaction is initiated during each Fmoc‐deprotection step that typically entails 20% piperidine (**1**) in DMF. Piperidine (pK_a_ = 11.1 of the corresponding acid) is a nucleophilic base which promotes not only Asi formation, but also nucleophilic attack onto the formed imide to form piperidides. The nonnucleophilic base 1,8‐diazabicyclo[5.4.0]undec‐7‐ene (**26**, DBU; pKa of conjugate acid = 13.5) is an alternative reagent for rapid Fmoc‐deprotection. However, its high basicity promotes Asi formation to a greater extent, often rendering it unsuitable for Asi‐prone sequences.^[^ ^30^ ^]^

**Figure 4:**
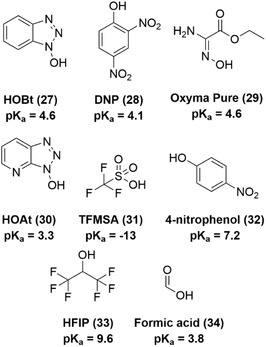
Overview of additives used during Fmoc‐deprotection and their corresponding pKa.

In 1994, it was already observed that the addition of 0.1 M hydroxybenzotriazole (**27**, HOBt, pK_a_ = 4.6) or 2,4‐dinitrophenol (**28**, DNP, pK_a_ = 4.1) reduced Asi formation significantly (**Figure** **4**).^[^ ^90^ ^]^ In this case, the reduction in aspartimide formation is likely due to the ability of HOBt and DNP to buffer the deprotection environment and reduce undesired deprotonation of the backbone amide. However, prolonged presence of weak acids can compromise acid‐sensitive resin‐linkers such as 2‐chlorotrityl chloride (2‐CTC),^[^ ^42^ ^]^ potentially causing premature cleavage from the solid support.

More recently, ethyl 2‐cyano‐2‐(hydroxyimino)acetate (**29**,Oxyma Pure) has been employed in Fmoc‐deprotection cocktails and has proven to be even more effective at suppressing Asi formation than both HOBt and 7‐aza‐1‐hydroxybenzotriazole (**30**, HOAt) at 1 M concentration.^[^ ^91^ ^]^ Various acids were screened by Michels et al., including trifluoromethanesulfonic acid (**31**, TFMSA, pK_a_ = −13), 4‐nitrophenol (**32**, pK_a_ = 7.2), and hexafluoro isopropanol (**33**, HFIP, pK_a_ = 9.6).^[^ ^92^ ^]^ Among these acids, TFMSA caused side‐products in addition to aspartimide‐related compounds, while HFIP had no significant effect on suppressing Asi formation. Instead, 5% formic acid (**34**, pK_a_ = 3.8) was sufficient to reduce Asi formation during the synthesis of peptide PTH by 90%.^[^ ^92^ ^]^

**Figure 5:**
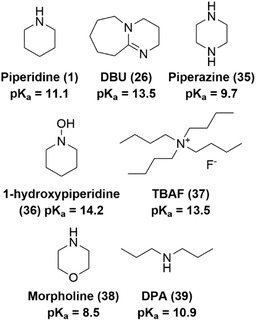
Overview of bases used in Fmoc‐deprotection and the corresponding pKa values of either their conjugated acid or their acidic proton.

An alternative approach involves altering the base itself (**Figure** **5**). Wade et al. introduced piperazine (**35**), 1‐hydroxypiperidine (**36**), and tetrabutylammonium fluoride (**37**) as alternatives to piperidine.^[^ ^93^ ^]^ The impact on Asi formation was determined using the scorpion toxin II model hexapeptide. 1‐hydroxypiperidine and piperazine showed significant improvement over piperidine, with around 60% of the peptide obtained compared to 16%. TBAF, in contrast, did not show improvements.^[^ ^93^ ^]^

While morpholine (**38**) was already employed during deprotection procedures of PNAs,^[^ ^94^ ^]^ it was later described by Mthembu et al. as a green alternative to piperidine with less Asi‐related byproducts.^[^ ^95^ ^]^ For example, using ≈50% morpholine in DMF resulted in minimal Asi formation (1.2% at rt and 4.3% at 45 °C), in contrast to piperidine (9.2% at rt and >70% at 45 °C) in the synthesis of the toxinII model peptide.^[^ ^95^ ^]^ Additionally, the use in stapled peptides often requires the use of OAllyl groups as aspartic acid side chain protecting groups, but when using piperidine, Asi formation is inevitable. Some reports suggest that morpholine minimizes the formation of aspartimide, allowing successful synthesis of stapled peptides.^[^ ^96^ ^]^ More recently, dipropylamine (**39**, DPA) was shown to be effective for Fmoc‐deprotection.^[^ ^97^ ^]^ This unregulated, nonodorous, and more inexpensive chemical considerably reduces Asi formation. However, when synthesizing more challenging peptide syntheses yields are generally lower.^[^ ^97^ ^]^

### 4.2 N^α^ Protecting Groups

**Figure 6:**
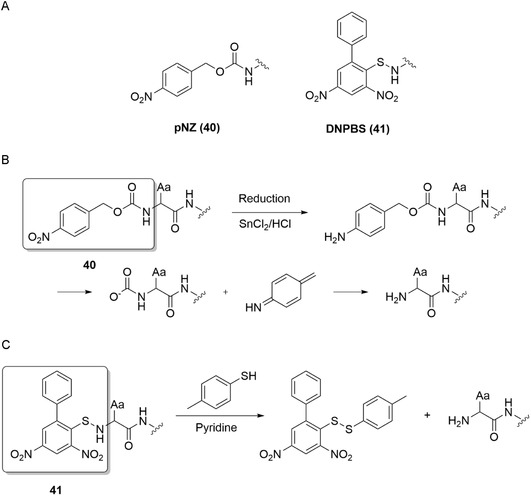
A) overview of N^α^ protecting groups. B) Removal of pNZ (**40**) group C) Removal of DNPBS (**41**) group. Aa = any amino acid.

Given that optimized Boc‐chemistry SPPS can already be used to synthesize peptides without aspartimide formation, it suggests an alternative approach to eliminate this side reaction, namely, by avoiding the use of strong base during deprotection in Fmoc chemistry SPPS. In 2005, such a concept was first introduced by Isidro‐Llobet et al. by using an alternative temporary N^α^ protecting group.^[^ ^98^ ^]^ The use of *p*‐nitrobenzyloxycarbonyl (**40**, pNZ) was beneficial since cleavage was performed under mild conditions with SnCl_2_ and a catalytic amount of HCl (**Figure** **6**). pNZ‐AAs were obtained via the coupling of a free amino acid with pNZ‐N_3_.^[^ ^98^ ^]^ No formation of Asi nor β‐peptides was observed when using a hybrid strategy of pNZ and Fmoc chemistry compared to using exclusively Fmoc chemistry on model peptide H‐Ala‐Orn‐Asp‐Gly‐Tyr‐Ile‐NH_2_. Notably, pNZ is orthogonal to Fmoc, making it a beneficial side‐chain protecting group for Lys and Orn.^[^ ^99^ ^,^ ^100^ ^]^ This synthetic methodology has also been used to obtain ester‐linked branched PNA‐peptide conjugates, expanding the toolbox for PNA probes and PNA‐encoded chemical libraries.^[^ ^101^ ^]^

Zhou et al. have proposed a new amine protecting group with 2,4‐dinitro‐6‐phenyl‐benzene sulfenyl (**41**, DNPBS).^[^ ^102^ ^]^ Sidechain‐protected amino acids can then be treated with trimethylsilyl chloride (TMSCl) and 4‐methyl morpholine in CH_2_Cl_2_/CH_3_CN to obtain DNPBS‐protected amino acids in high yields. The S—N bond can be quantitatively converted into the free amine under mild conditions (1 M *p*‐toluenethiol in pyridine).^[^ ^102^ ^]^ However, one challenge that may be encountered is its maximum residue length of 10 residues on polystyrene resin. Although polyethylene glycol‐based ChemMatrix resin allows for longer sequences, the coupling efficiency remains lower than that of standard Fmoc chemistry SPPS. Hence, a hybrid strategy was proposed in which two amine protecting groups are employed to complement each other's limitations. Recent developments have been made by the same group in optimizing the DNPBS group by systematically modifying the nitrobenzenesulfenyl scaffold to improve both chemical stability and thiol‐mediated deprotection kinetics.^[^ ^103^ ^]^

## 5 Conclusion & Future Perspectives

Ester‐based β‐carboxyl protecting groups with sterically more demanding groups have been extensively studied and have been shown to result in less Asi formation. Despite feasible incorporation and workflow adaptation, these esters are still sufficiently electrophilic to undergo Asi formation to an extent. This prompted peptide chemists to focus on developing new non‐ester‐based masking strategies for the aspartic acid side chain that eliminate these challenges. Stable C—C ylide‐based, and C—N hydrazides are promising examples of aspartic acid synthons which eliminate Asi formation due to adjusting the electrophilic nature of the β‐carboxyl, but the necessary use of oxidative conditions and copper salts, respectively, limits their application. Extending the ylide‐based protecting groups with CyPY eliminates the use of an oxidative halogen source. Instead, aqueous acid facilitates deprotection while offering compatibility with oxidative‐prone amino acids.

Backbone protecting groups are beneficial because Asi formation is completely diminished. But coupling efficiency drops when using these stand‐alone, making their incorporation into the standard workflow challenging. While some dipeptide building blocks are commercially available, the lack of combinations and relatively high prices make them less viable for noncanonical amino acids and as a universal solution. Backbone‐protecting groups with favorable *O*,*N*‐acylation kinetics have potential, as they can be automated on‐resin. However, the use of carcinogenic reducing agents like CrCl_2_ is not optimal.

The use of N‐hydroxylamines or other weak acids for buffering the strong basic environment during Fmoc‐removal is attractive as these additives do not alter standard Fmoc‐SPPS procedures. However, elongated peptides will still endure many Fmoc‐deprotection cycles, which will end up with Asi formation. The use of different N^α^ protected amino acids, like pNZ or DNPBS, abolishes basic deprotection conditions, but by the cost of coupling efficiency with longer peptide sequences.

It is also important to consider compatibility with emerging synthetic tools in peptide chemistry, such as bioorthogonal chemistry, unnatural amino building blocks, and flow chemistry.^[^ ^104^, ^105^, ^106^ ^]^ In addition, the construction of molecular complexity, such as cyclic peptides, often requires post‐SPPS modifications, which must be compatible with the chosen protecting group strategy.^[^ ^107^, ^108^ ^]^

To conclude, aspartimide formation has long posed a major challenge in Fmoc‐based SPPS, with ester‐based protecting groups often proving insufficiently effective (Section2). In contrast, the use of backbone‐protecting groups and the recently reported non‐ester‐based aspartic acid side‐chain masking groups (Section3) have been shown to completely suppress Asi formation and will likely guide future developments in the field. Building on these advances, further work is needed to make these approaches broadly accessible and applicable to routine peptide synthesis.

## Conflict of Interest

The authors declare no conflict of interest.
